# Optimizing alternate replacement strategy of *k*-out-of-*n*: F systems with common-mode degradation: An application to railway grid

**DOI:** 10.1371/journal.pone.0322001

**Published:** 2025-05-22

**Authors:** Liying Wang, Mingjie Ding, Boshi Liu, Qingan Qiu

**Affiliations:** 1 Department of Mathematics and Physics, Shijiazhuang Tiedao University, Shijiazhuang, China; 2 School of Management, Beijing Institute of Technology, Beijing, China; Aalto University, FINLAND

## Abstract

Under the grid management framework for railway lines, each rail grid consists of several adjacent steel rails. The degradation processes of the steel rails within a grid are characterized by common-mode deterioration due to their similar spatial locations and accumulated gross tonnages. Additionally, in line with the railway repair and maintenance regulations, comprehensive replacements of rail grids are prohibited during the summer months, and similar restrictions apply in winter. To effectively minimize the operating and maintenance costs associated with a rail grid over a one-year period, this study develops a *k*-out-of-*n*: F system model that incorporates common-mode degradation. A dynamic and alternate replacement strategy is proposed, focusing on both system-level and component-level interventions. The degradation of each steel rail is modeled using a Gamma process, while the dependence structure of the system is characterized through a copula function. The maintenance model is structured as a Markov Decision Process (MDP). To address the MDP problem, approximate and analytical expressions for discretized state transition probabilities within the multiple-component system are derived. These expressions are determined using the copula function, and an algorithm is designed to construct the corresponding transition probability matrix. The monotonicity of value functions is also explored. A numerical example is provided to demonstrate the feasibility and effectiveness of the proposed model.

## 1. Introduction

Numerous industrial systems are comprised of multiple components that must work together to efficiently and effectively achieve their objectives. The interdependence among these components is unavoidable [1,2]. Ignoring component interdependence can lead to overestimating the reliability of multi-component repairable systems and result in impractical maintenance decisions [3]. Consequently, integrating the interdependence has become crucial in reliability analysis and maintenance strategy optimization. The advances in sensing and monitoring technologies have increased interest in condition-based maintenance (CBM) [4,5]. However, optimizing CBM strategies for systems with interdependent components presents significant challenges in both academic and industrial contexts. This is due to the complex dependence structures, multiple failure effects, and the need to balance maintenance costs with the system reliability [6].

**Fig 1:**
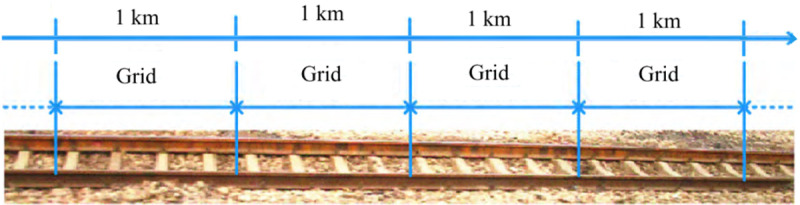
Diagrammatic sketch of railway grid.

This study is motivated by the operation and maintenance of railway rails in China, where steel rails represent a vital component of the railway track infrastructure. As time progresses, the performance of these rails gradually deteriorates, making regular maintenance and replacement essential to ensure the safety, smoothness, and comfort of train journeys. In China, a comprehensive grid management strategy for railway lines is currently being rolled out. This approach categorizes railway lines into adjacent segments, each spanning 1 km in length, referred to as a “grid”. As shown in Fig 1, a grid includes several steel rails. The condition of individual steel rails can be classified into four states: normal, minorly defective, severely defective, or broken [7]. The state of a rail grid is defined by the number of severely defective or broken rails it contains; if this count exceeds a predetermined threshold, the entire grid is replaced. Both the threshold and the number of defective rails are influenced by geographical factors specific to the region. To model this phenomenon, rail grids can be conceptualized as a *k*-out-of-*n*: F system.

According to [8], several heterogeneous factors contribute to the degradation of steel rails, including spatial characteristics such as narrow-radius curves and steep gradients, as well as cumulative gross tonnage. Given that rails within a grid experience the same accumulated tonnage, their degradation is often subject to common-mode failure. Additionally, rails located in sections with tight curves or steep slopes exhibit similar common-mode dependence due to their shared environmental conditions. Moreover, China’s railway repair and maintenance regulations [9,10] prohibit comprehensive replacements of rail grids to mitigate the risks of rail expansion during hot summer months, a guideline that remains applicable in winter as well. Conversely, replacement activities at the component level are relatively more flexible. In this context, this paper proposes a novel *k*-out-of-*n*: F repairable system model that accounts for common-mode deterioration. Additionally, it discusses various alternate replacement strategies at both the system and component levels, providing insights that could enhance the efficacy of maintenance practices within the rail industry.

## 2. Related literature

### 2.1. Maintenance optimization for systems with common-mode deterioration

The literature discusses various forms of dependence, including stochastic, economic, and structural [11–14]. Among these, stochastic dependence has garnered the most attention due to its complexity. Stochastic dependence in maintenance modeling can be categorized into three types based on different characteristics: failure interactions, load sharing, and common mode deterioration [6,15,16]. Methods for describing stochastic dependence typically involve multivariate joint distribution models, copula-based models, and degradation rate interaction models [17].

Our work focuses on multi-component systems with common-mode deterioration. Common-mode deterioration occurs when components experience simultaneous degradation due to similar operating conditions. A key feature of this phenomenon is that the acceleration or deceleration of degradation for each component typically transpires concurrently. Various studies have addressed the reliability analysis and optimization of operation and maintenance for multiple-component systems experiencing common-mode deterioration [18]. Approaches such as multivariate subordinator processes [19,20], Wiener processes with positive correlation coefficients have been employed to model the dependence structures of these systems [21].

### 2.2. Reliability modeling based on copula functions

The copula-based method, introduced by Sklar in 1959, has found extensive application in reliability modeling [22–24]. This approach effectively separates the dependence structure of a set of random variables from their marginal distribution functions, facilitating easier parameter estimation. Additionally, it permits the definition of various multivariate distributions with consistent dependence structures by allowing different marginal distributions, thus accommodating complex dependency structures such as nonlinear dependencies and tail dependencies [25]. The function that links multiple distributions is known as an ordinary copula, which has been utilized to quantify the dependence structure of component lifetimes [26–30] and component deterioration increments over specified time intervals [31,32].

To address the limitations posed by ordinary copulas, particularly their reliance on time and the potentially unbounded number required for modeling stochastic processes, Tankov extended copula theory to include Lévy copulas. Lévy copulas quantify the dependence structure among processes using finite and time-independent parameters and allow for distinct marginal processes. For instance, [33] employed Lévy copulas to characterize the common-mode degradation dependence between two components, proposing a novel condition-based maintenance strategy. Building on this, [6] utilized nested Lévy copulas to quantify the hierarchical common-mode degradation dependence structure of multiple components and explored a new inspection and replacement strategy. In these strategies, the degradation information for unmonitored components at inspection epochs is updated based on the most recently monitored component and the conditional copula functions. By leveraging stochastic dependence, these strategies have been shown to be more efficient.

Regarding steel rails, univariate Gamma processes and bivariate Gamma subordinator processes have been used to model wear degradation [34,35]. Additionally, discussions on the optimization of operations and maintenance for steel rails have also been addressed, highlighting the significance of effective strategies in prolonging the life and reliability of these critical infrastructure components.

The main contributions of this paper are as follows: (1) A *k*-out-of *n*: F repairable system with common-mode deterioration dependence is constructed to model the damage processes of rail grids. According to the reports from the professional fields, the common-mode dependence among steel rails in a grid is characterized by a Lévy copula. (2) Based on the railway lines’ repair and maintenance management rules over one year, an alternate replacement strategy of system level and component level is proposed. The alternate replacement strategy can be used to develop annual maintenance and repair plan of rail grids. Markov decision process (MDP) model under finite stages is established to get the optimal strategy. (3) To solve the MDP problem, analytical and approximate expressions of discretized states transition probabilities for the multiple component system are obtained. The expressions can be determined by the Lévy copula function and an algorithm is designed to get the matrix.

The remainder of the paper is organized as follows. Modelling assumptions and the alternate replacement strategy are given in Section 2. In Section 3, the system states are discretized. The analytical expressions of the system state transition probabilities are discussed. To get the optimal alternate replacement strategy of system level and component level, Markov decision process model of four stages is established in Section 4. State transition probability matrix algorithm and backward dynamic programming algorithm are designed. The monotonicity of value functions is also discussed in this section. In Section 5, a numerical example is given to illustrate the efficiency of the model. Sensitivity analysis of dependence strength and cost-related parameters is also performed.

## 3. Modelling assumptions and the alternate replacement strategy

### 3.1. Modelling *k*-out-of-*n* systems with common-mode deterioration

We consider a k -out-of- n: F system which fails if and only if at least k of the n components fail. Let $X_{t}^{i}(i=1,\cdots ,n)$ denote the degradation state of component i at time t. The degradation increment of component i (i=1,⋯,n) from time s to t (s<t), $X_{t}^{i}-X_{s}^{i}$ is Gamma distributed and the initial state $X_{0}^{i}=0\;(i=1,\cdots ,n)$. Component i (i=1,⋯,n) fails if its degradation state exceeds the failure threshold L.

The random vector $\left\{X_{t},t\geq 0\}=\{X_{t}^{1},\cdots ,X_{t}^{n}\right\}$ is used to denote the system degradation state at time t. Assume that the system state increments $X_{t}-X_{s}(0\leq s<t)$ are independent and time homogeneous. The degradation processes of all components are common-mode dependence and the dependence structure can be quantified by a Lévy copula function. According to the limit theorem of Lévy copulas, given time t, we can find the appropriate ordinary copula $C_{t}(⬝,\cdots ,⬝)$, such that the joint distribution of $X_{t}$ [6]


$$H_{X_{t}}(y_{1},\cdots ,y_{n})=C_{t}(F_{X_{t}^{1}}(y_{1}),\cdots ,F_{X_{t}^{n}}(y_{n})),$$

where $F_{X_{t}^{i}}(y_{i},(i=1,\cdots ,n)$ is the marginal distribution of $X_{t}^{i}$ and $C_{t}(⬝,\cdots ,⬝)$ is dependent on t.

**Fig 2:**
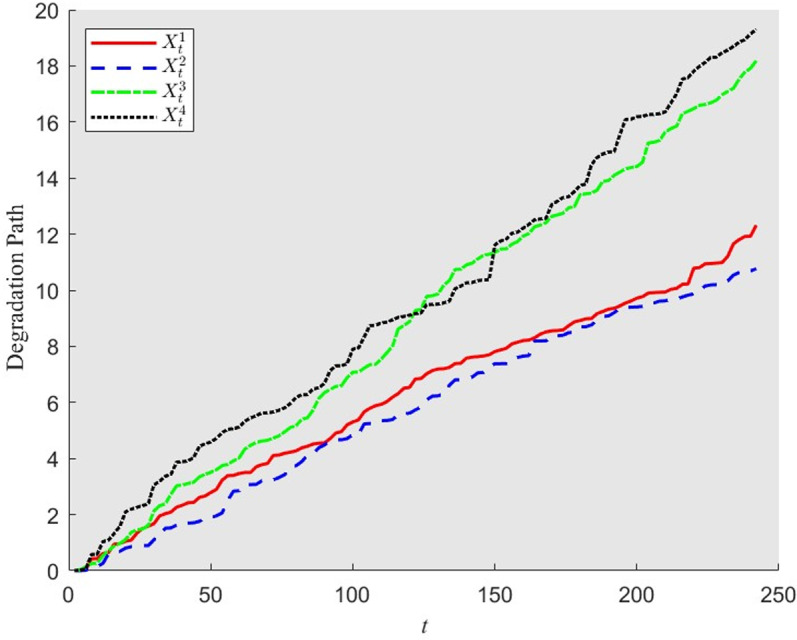
A deterioration path of four component system with copula dependence.

Fig 2 shows a sample of deterioration path for a four-component system which is modeled by Gamma processes with copula dependence. We can observe that the four components degrade in the similar trend. Furthermore, the degradation processes of components 1 and 2 are much closer and those of components 3 and 4 are no significant difference.

The main reasons that copula functions are chosen to quantify the common-mode dependence among steel rails in a grid are as follows: (1) The copula method has significant convenience in parameter estimation and great flexibility in describing dependency relationships. (2) Massive literature has utilized copula functions to model the dependence structures among components’ degradation processes, which is stated in Section 2.2. (3) In [6], copula functions are used to characterize the dependence structures of four wheels’ degradation processes in a vehicle. Rolling friction and sliding friction are the main causes for the degradations of wheels and steel rails. Thus the degradation mechanism of the rail gird is similar to that of the wheel system.

### 3.2. The alternate replacement strategy with system level and component level

The track inspection cars are capable of assessing the condition of steel rails, including wear levels, deformation, and any abnormal noises. At the start of each season, managers should plan the annual maintenance activities in accordance with maintenance regulations based on the condition of each rail grid as reported by the inspection cars. To effectively schedule the maintenance activities of a rail grid over a one-year period, we set the time inspection interval δ to be one season and consequently the terminating time of one-year can be denoted by 4Kδ within the lifecycle of a rail grid. Then we employ MDP to model the alternate replacement decisions with the objective of minimizing the cost over [4Kδ,4Kδ+4δ) (the cost over one-year).

The failures of the system and components are non-self-announcing and the system is monitored periodically with time interval δ. We consider a four-stage decision problem with the terminating time given by 4Kδ. The inspection is merely an information-taking action that reveals perfectly the degradation level of all components. After the inspection, the alternate replacement strategy of system level and component level is performed as follows.

(1) At time 4Kδ, if the system fails, it will be replaced in entirety; otherwise, the system can be replaced in entirety or doing nothing.
(2) At time 4Kδ+δ, if the system fails, it will be replaced in entirety; otherwise, every component can be replaced or doing nothing.
(3) At time 4Kδ+2δ, the replacement strategy of system level is the same to that at time 4Kδ. At time 4Kδ+3δ, the replacement strategy of component level is the same to that at time 4Kδ+δ.
(4) The time for inspections and replacements is negligible. The cost for the replacement of component i(i=1,⋯,n) is $c_{i}$. The system replacement in entirety or replacements of components will incur a constant set-up cost $C_{s}$. Given the system degradation state after the replacements or doing nothing at an inspection epoch $\chi =(x_{1},\cdots ,x_{n})$, let $C_{pc}(\chi )=C_{f}g(\frac{l_{\chi }}{n})$ be the penalty cost of system failure over an inspection interval, where $C_{f}$ is the fundamental penalty cost of system failure, $l_{\chi }$ is the number of failed components. We assume that the function g increases with $\frac{l_{\chi }}{n}$.

## 4. Discretizing the system states and the system state transition probabilities

### 4.1. Discretizing the system states

A discrete space state is often required in order to solve an MDP and it is practical in engineering practice. In this section, the states of the multiple component system will be discretized and the action spaces of the alternate replacement strategies are also given [31,36,37].

**Fig 3:**
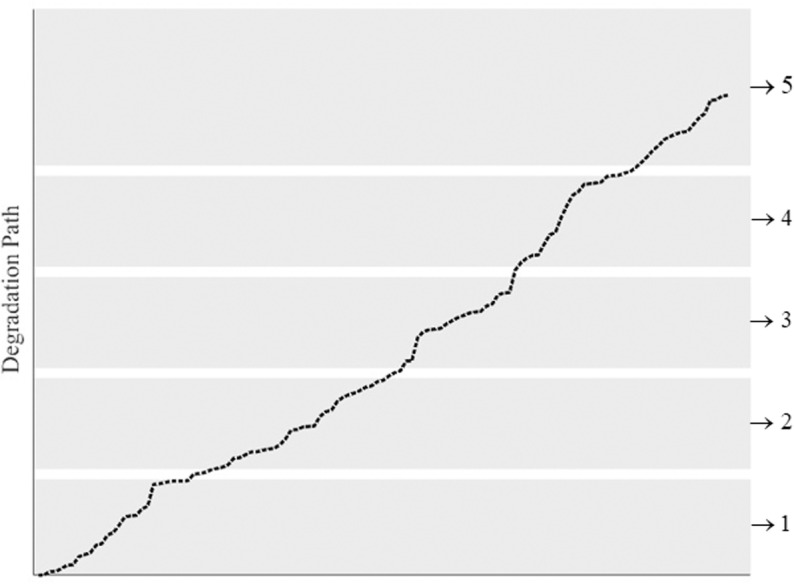
Discretization of the degradation characteristic for one dimension.

Let Δd be the discretization interval of the continuous degradation components. The interval (0,L) can be discretized into N equally sized intervals. Component i(i=1,⋯,n) is said in state $x_{i}$ if and only if $(x_{i}-1Deltad\leq X_{t}^{i}<x_{i}\Delta d,x_{i}=1,\cdots ,N$ and the failed state N+1 corresponds to the interval [NΔd,+∞). After the discretization, the state space of each component S={1,⋯,N,N+1}. The illustration of state discretization for one component is shown in Fig 3. In Fig 3, the continuous states of one component are discretized into 5 states and 5 is the failed state. The failure threshold is equal to 4Δd.

Let


$$F_{X_{t}^{i}}(x_{i}triangleqP\{X_{t}^{i}\leq x_{i}\Delta d\},x_{i}=1,\cdots ,N,\;i=1,\cdots ,n,$$

then


$$F_{X_{t}^{i}}(x_{i})=\frac{\Gamma (\alpha_{i}t,\beta_{i}x_{i}\Delta d)}{\Gamma (\alpha_{i}t)},$$

where $\Gamma (\alpha_{i}t,\beta_{i}x_{i}\Delta d)=\int_{0}^{\beta_{i}x_{i}\Delta d}u^{\alpha_{i}t-1}e^{-u}du$ is the incomplete Gamma function. Let χ be the discretized system state space, then $\chi =(x_{1},\cdots ,x_{n}),x_{i}\in \{1,\cdots ,N\},i=1,\cdots ,n$. For every $\chi =(x_{1},\cdots ,x_{n}),x_{i}\in \{1,\cdots ,N\},i=1,\cdots ,n$, from the modeling assumptions and the properties of joint distribution functions [24]


$$P\{X_{t}=\chi \}=\sum_{J\in {\left\{0,1\right\}}^{n}}{\left(-1\right)}^{\sum_{k=1}^{n}j_{k}}C_{t}(F_{X_{t}^{1}}{\left(x_{1}-1\right)}^{j_{1}}F_{X_{t}^{1}}{\left(x_{1}\right)}^{1-j_{1}},\cdots ,F_{X_{t}^{n}}{\left(x_{n}-1\right)}^{j_{n}}F_{X_{t}^{n}}{\left(x_{n}\right)}^{1-j_{n}})$$ (1)

Let $a_{j}=(a_{j_{1}},\cdots ,a_{j_{n}})$ denote a maintenance action for the system, where $a_{j_{i}}=1$ and $a_{j_{i}}=0$ stand for the replacement of component i(i=1,⋯,n) and doing nothing on it, respectively. According to the modeling assumptions, under the replacement strategy of system level, the action space ${𝒜}_{s}=\{{𝐚}_{1},{𝐚}_{0}\}$, where $a_{1}=(1,\cdots ,1)$, $a_{0}=(0,\cdots ,0)$ and the action space for the replacement strategy of component level, ${𝒜}_{u}=\{a_{j}|a_{j}=(a_{j_{1}},\cdots ,a_{j_{n}}),\;a_{j_{i}}\in \{0,1\},j=1,\cdots ,2^{n}\}$.

For $\chi =(x_{1},\cdots ,x_{n})$, let $M^{a}(\chi )$ be the system state after a maintenance action $a=(a_{1},\cdots ,a_{n})$, then


$$M^{a}(\chi )=(x_{1}^{1-a_{1}},x_{2}^{1-a_{2}},\cdots ,x_{n}^{1-a_{n}}).$$

Specially, $M^{a_{0}}(\chi )=\chi$, $M^{a_{1}}(\chi )=(1,\cdots ,1)$.

### 4.2. State transition probabilities

Let $X_{l\delta }$ (l=1,2,3,4) be the state of the system at the 4K+l−1 th inspection. When no maintenance actions are taken, the state transition probability matrix over an inspection interval is denoted as $R≜\{R(\chi^{\prime }|\chi );\chi \in \chi ,\;\chi^{\prime }\in \chi \}$, where


$$R(\chi^{\prime }|\chi )=P\{X_{(l+1)\delta }=\chi^{\prime }|X_{l\delta }=\chi \},\forall \chi ,\chi^{\prime }\in \chi .$$

Due to the independent and time homogeneous increment degradation process, R is also time homogeneous.

In the MDP, the transition probability is usually approximated by [31,32]


$$\begin{array}{cc} & R(\chi^{\prime }|\chi )=P\{({x^{\prime }}_{1}-1)\Delta d\leq X_{(l+1)\delta }^{1}<{x^{\prime }}_{1}\Delta d,\cdots ,({x^{\prime }}_{n}-1)\Delta d\leq X_{(l+1)\delta }^{n}<{x^{\prime }}_{n}\Delta d\\ & \;|X_{l\delta }=((x_{1}-1)\Delta d,\cdots ,(x_{n}-1)\Delta d)\},\forall \chi ,\chi^{\prime }\in \chi \;, \end{array}$$ (2)

i.e., we assume each component is at the left endpoint of continuous degradation interval at the inspection epoch. In the literatures, Monte Carlo simulation is usually used to estimate the probabilities in Equation 2. In this paper, analytical expressions of $R(\chi^{\prime }|\chi )$ under different cases will be discussed.

**Case 1**: $\chi =(x_{1},\cdots ,x_{n}),$ $\chi^{\prime }=({x^{\prime }}_{1},\cdots ,{x^{\prime }}_{n})(x_{i},{x^{\prime }}_{i}\in \{1,\cdots ,N,N+1\},i=1,\cdots ,n)$ and there exists at least one pair $x_{i},\;\;{x^{\prime }}_{i}$ (i=1,⋯,n) such that $x_{i}>{x^{\prime }}_{i}$.

If there exists at least one pair $x_{i},\;{x^{\prime }}_{i}$ such that $x_{i}>{x^{\prime }}_{i}$, then at least one component’s degradation state in the system reduces over δ time. Form the increasing property of Gamma process, the event is impossible when no maintenance action is taken, hence $R(\chi^{\prime }|\chi )=0$ under this case. Therefore, R is an upper triangular matrix.

**Case 2**: $\chi =(x_{1},\cdots ,x_{n}),\chi^{\prime }=({x^{\prime }}_{1},\cdots ,{x^{\prime }}_{n})(x_{i},{x^{\prime }}_{n}\in \{1,\cdots ,N\},i=1,\cdots ,n)$ and for every i∈{1,⋯,n}, $x_{i}<{x^{\prime }}_{i}$.

Under this case, all components are in working states after and before the transition. Furthermore, all components degrade to worse states after the transition. From Equation 2,


$$\begin{array}{cc} & R(\chi^{\prime }|\chi )=P\{({x^{\prime }}_{1}-1)\Delta d\leq X_{(l+1)\delta }^{1}<{x^{\prime }}_{1}\Delta d,\cdots ,({x^{\prime }}_{n}-1)\Delta d\leq X_{(l+1)\delta }^{n}<{x^{\prime }}_{n}\Delta d\\ & \;\;\;|X_{l\delta }^{1}=(x_{1}-1)\Delta d,\cdots ,X_{l\delta }^{n}=(x_{n}-1)\Delta d\}. \end{array}$$ (3)

Since the increment processes of the system states are the independent and time homogeneous, Equation 3 can be rewritten as

$R(\chi^{\prime }|\chi )=P\{({x^{\prime }}_{1}-x_{1}Deltad\leq X_{\delta }^{1}<({x^{\prime }}_{1}-x_{1}+1)\Delta d,\cdots ,({x^{\prime }}_{n}-x_{n})\Delta d\leq X_{\delta }^{n}<({x^{\prime }}_{n}-x_{n}+1)\Delta d\}.$

From Equation 1, the above equation can be expressed as


$$R(\chi^{\prime }|\chi )=\sum_{J\in {\left\{0,1\right\}}^{n}}{\left(-1\right)}^{\sum_{k=1}^{n}j_{k}}C_{\delta }\{[F_{X_{\delta }^{1}}{\left({x^{\prime }}_{1}-x_{1}\right)}^{j_{1}}F_{X_{\delta }^{1}}{\left({x^{\prime }}_{1}-x_{1}+1\right)}^{1-j_{1}}],\cdots ,[F_{X_{\delta }^{n}}{\left({x^{\prime }}_{n}-x_{n}\right)}^{j_{n}}F_{X_{\delta }^{n}}{\left({x^{\prime }}_{n}-x_{n}+1\right)}^{1-j_{n}}]\}.$$

This paper only involves the analysis of transition probabilities over δ time interval, for simplicity, we denote $C_{\delta }$ as C and $F_{X_{\delta }^{i}}(i=1,\cdots ,n)$ as $F_{i}(i=1,\cdots ,n)$, respectively.

In summary, under case 2


$$R(\chi^{\prime }|\chi )=\sum_{J\in {\left\{0,1\right\}}^{n}}{\left(-1\right)}^{\sum_{k=1}^{n}j_{k}}C\{[F_{1}{\left({x^{\prime }}_{1}-x_{1}-1\right)}^{j_{1}}F_{1}{\left({x^{\prime }}_{1}-x_{1}\right)}^{1-j_{1}}],\cdots ,[F_{n}{\left({x^{\prime }}_{n}-x_{n}-1\right)}^{j_{n}}F_{n}{\left({x^{\prime }}_{n}-x_{n}\right)}^{1-j_{n}}]\}.$$ (4)

If the failed state is 6, then χ=(1,2,3,4) and $\chi^{\prime }=(2,\;3,\;4,\;5)$ meet with the conditions of case 2 and there are 16 terms on the right side of Equation 4. Furthermore J is four-dimensional vector whose elements are 0 or 1. For $J=(j_{1},j_{2},j_{3},j_{4})$, the term corresponds to it is $C(F_{1}{\left(1\right)}^{j_{1}}F_{1}{\left(2\right)}^{1-j_{1}},F_{2}{\left(1\right)}^{j_{2}}F_{2}{\left(2\right)}^{1-j_{2}},F_{3}{\left(1\right)}^{j_{3}}F_{3}{\left(2\right)}^{1-j_{3}},F_{4}{\left(1\right)}^{j_{4}}F_{4}{\left(2\right)}^{1-j_{4}})$.

**Case 3**: $\chi =(x_{1},\cdots ,x_{n})$, $\chi^{\prime }=({x^{\prime }}_{1},\cdots ,{x^{\prime }}_{n})$, $x_{i},{x^{\prime }}_{i}\in \{1,\cdots ,N\},i=1,\cdots ,n$, where $x_{i_{h}}={x^{\prime }}_{i_{h}}$ $(h=1,\cdots ,m),\;\;x_{i_{l}}<{x^{\prime }}_{i_{l}}(l=m+1,\cdots ,n).$

Under case 3, within δ time interval, the state of component $i_{h}\;(h=1,\cdots ,m)$ remains unchanged and in working state and the other n−m components degrade to worse and working states.

By the law for the intersection of events, under case 3,


$$\begin{array}{cc} & R(\chi^{\prime }|\chi )=P(0\leq X_{\delta }^{i_{1}}<\Delta d,\cdots ,0\leq X_{\delta }^{i_{m}}<\Delta d,({x^{\prime }}_{i_{m+1}}-x_{i_{m+1}})\Delta d\leq X_{\delta }^{i_{m+1}}<({x^{\prime }}_{i_{m+1}}-x_{i_{m+1}}+1)\Delta d,\\ & \;\cdots ,({x^{\prime }}_{i_{n}}-x_{i_{n}})\Delta d\leq X_{\delta }^{i_{n}}<({x^{\prime }}_{i_{n}}-x_{i_{n}}+1)\Delta d)\}. \end{array}$$

From Equation 1,


$$\begin{array}{cc} & R(\chi^{\prime }|\chi )=\sum_{J\in {\left\{0,1\right\}}^{n}}{\left(-1\right)}^{\sum_{k=1}^{n}j_{k}}C_{S_{i_{m}}⋃{\bar{S}}_{i_{m}}}(F_{i_{1}}{\left(0\right)}^{j_{1}}F_{i_{1}}{\left(1\right)}^{1-j_{1}},\cdots ,F_{i_{m}}{\left(0\right)}^{j_{m}}F_{i_{m}}{\left(1\right)}^{1-j_{m}}),\\ & \;F_{i_{m+1}}{\left({x^{\prime }}_{i_{m+1}}-x_{i_{m+1}}\right)}^{j_{m+1}}F_{i_{m+1}}{\left({x^{\prime }}_{i_{m+1}}-x_{i_{m+1}}+1\right)}^{1-j_{m+1}},\cdots ,F_{i_{n}}{\left({x^{\prime }}_{i_{n}}-x_{i_{n}}\right)}^{j_{n}}F_{i_{n}}{\left({x^{\prime }}_{i_{n}}-x_{i_{n}}+1\right)}^{1-j_{n}}), \end{array}$$ (5)

where $S_{i_{m}}=\{i_{1},\cdots ,i_{i_{m}}\},\;{\bar{S}}_{i_{m}}=\{i_{m+1},\cdots ,i_{n}\}$. By setting the $i_{l}$ th (l=1,⋯,n) variable in copula function C as the value shown in Equation 5, we can obtain $C_{S_{i_{m}}⋃{\bar{S}}_{i_{m}}}$.

It is known from the nonnegative property of $Xi_{h}(h=1,\cdots ,m)$ that $F_{i_{h}}(0)=0(h=1,\cdots ,m)$, hence, for $j_{h}=1$, $F_{i_{h}}{\left(0\right)}^{j_{h}}F_{i_{h}}{\left(1\right)}^{1-j_{h}}=0$. Consequently, for $J=(j_{1},\cdots ,j_{m},j_{m+1},\cdots ,j_{n})$, from the property of copula functions, if there exists at least one $j_{h}=1(h=1,\cdots ,m)$, $C_{S_{i_{m}}⋃{\bar{S}}_{i_{m}}}$ in Equation 5 will be zero. Furthermore, if $j_{h}=0\;(h=1,\cdots ,m)$, $F_{i_{k}}{\left(0\right)}^{j_{k}}F_{i_{k}}{\left(1\right)}^{1-j_{k}}=F_{i_{k}}(1)$ and $\sum_{k=1}^{n}j_{k}=\sum_{k=m+1}^{n}j_{k}$. To sum up, under case 3,


$$\begin{array}{cc} & R(\chi^{\prime }|\chi )=\sum_{J\in {\left\{0,1\right\}}^{n-m}}{\left(-1\right)}^{\sum_{k=m+1}^{n}j_{k}}C_{S_{i_{m}}⋃{\bar{S}}_{i_{m}}}(F_{i_{1}}(1),\cdots ,F_{i_{m}}(1),\\ & \;F_{i_{m+1}}{\left({x^{\prime }}_{i_{m+1}}-x_{i_{m+1}}\right)}^{j_{m+1}}F_{i_{m+1}}{\left({x^{\prime }}_{i_{m+1}}-x_{i_{m+1}}+1\right)}^{1-j_{m+1}},\cdots ,F_{i_{n}}{\left({x^{\prime }}_{i_{n}}-x_{i_{n}}\right)}^{j_{n}}F_{i_{n}}{\left({x^{\prime }}_{i_{n}}-x_{i_{n}}+1\right)}^{1-j_{n}}). \end{array}$$ (6)

If the failed state is 6, then χ=(1,2,3,4), $\chi^{\prime }=(1,\;2,\;4,\;5)$ meet with the conditions of case 3. The states of components 1 and 2 are the same within δ time interval. According to Equation 6, there are 4 terms on the right side of it and J is two-dimensional vector whose elements are 0 or 1. Furthermore,


$$\begin{array}{cc} & R(\chi^{\prime }|\chi )=C(F_{1}(1),F_{1}(1),F_{3}(2),F_{4}(2)-C(F_{1}(1),F_{1}(1),F_{3}(2),F_{4}(1)\\ & \;\;-C(F_{1}(1),F_{1}(1),F_{3}(1),F_{4}(2)+C(F_{1}(1),F_{1}(1),F_{3}(2),F_{4}(2). \end{array}$$

**Case 4**: $\chi =(x_{1},\cdots ,x_{n})$, $\chi^{\prime }=({x^{\prime }}_{1},\cdots ,{x^{\prime }}_{n})$, where $x_{i_{j}}\in \{1,\cdots ,N\}(j=1,\cdots ,n)$, ${x^{\prime }}_{i_{h}}=N+1$ $\left(h=1,\cdots ,m),\;{x^{\prime }}_{i_{l}}\in \{1,\cdots ,N\}(l=m+1,\cdots ,n\right)$.

Under case 4, at the inspection epoch (4K+l−1delta, all components are in working states and at the inspection epoch (4K+ldelta, component $i_{h}\;(h=1,\cdots ,m)$ fails and the other n−m components remain in working states. According the modeling assumptions and Equation 2.


$$\begin{array}{cc} & R(\chi^{\prime }|\chi )=P\{(N+1-x_{i_{j}})\Delta d\leq X_{\delta }^{i_{h}}<+\infty ,h\in \{1,\cdots ,m\},\\ & \;({x^{\prime }}_{i_{l}}-x_{i_{l}})\Delta d\leq X_{\delta }^{i_{l}}<({x^{\prime }}_{i_{l}}-x_{i_{l}}+1)\Delta d,l\in \{m+1,\cdots ,n\}\}. \end{array}$$ (7)

From Equations 1, 7 can be rewritten as


$$\begin{array}{cc} & R(\chi^{\prime }|\chi )=\sum_{J\in {\left\{0,1\right\}}^{n}}{\left(-1\right)}^{\sum_{k=1}^{n}j_{k}}C(F_{i_{1}}{\left(N+1-x_{i_{1}}\right)}^{j_{1}}F_{i_{1}}{\left(+\infty \right)}^{1-j_{1}},\cdots ,F_{i_{m}}{\left(N+1-x_{i_{m}}\right)}^{j_{m}}F_{i_{m}}{\left(+\infty \right)}^{1-j_{m}},\\ & \;\;\;F_{i_{m+1}}{\left({x^{\prime }}_{i_{m+1}}-x_{i_{m+1}}\right)}^{j_{m+1}}F_{i_{m+1}}{\left({x^{\prime }}_{i_{m+1}}-x_{i_{m+1}}+1\right)}^{1-j_{m+1}},\cdots ,F_{i_{n}}{\left({x^{\prime }}_{i_{n}}-x_{i_{n}}\right)}^{j_{n}}F_{i_{n}}{\left({x^{\prime }}_{i_{n}}-x_{i_{n}}+1\right)}^{1-j_{n}}). \end{array}$$

Noting that $F_{i_{h}}(+\infty )=1,h=1,\cdots ,m,$ we have


$$\begin{array}{cc} & R(\chi^{\prime }|\chi )=\sum_{J\in {\left\{0,1\right\}}^{n}}{\left(-1\right)}^{\sum_{k=1}^{n}j_{k}}C_{S_{i_{m}}⋃{\bar{S}}_{i_{m}}}(F_{i_{1}}{\left(N+1-x_{i_{1}}\right)}^{j_{1}},\cdots ,F_{i_{m}}{\left(N+1-x_{i_{m}}\right)}^{j_{m}},\\ & \;F_{i_{m+1}}{\left({x^{\prime }}_{i_{m+1}}-x_{i_{m+1}}\right)}^{j_{m+1}}F_{i_{m+1}}{\left({x^{\prime }}_{i_{m+1}}-x_{i_{m+1}}+1\right)}^{1-j_{m+1}},\cdots ,F_{i_{n}}{\left({x^{\prime }}_{i_{n}}-x_{i_{n}}\right)}^{j_{n}}F_{i_{n}}{\left({x^{\prime }}_{i_{n}}-x_{i_{n}}+1\right)}^{1-j_{n}}). \end{array}$$ (8)

If the failed state is 6, then χ=(1,2,3,4), $\chi^{\prime }=(6,\;6,\;4,\;5)$ meet with the conditions of case 4. Components 1 and 2 transit to the failure state within δ time interval. According to Equation 8, there are 16 terms on its right side. For $J=(j_{1},j_{2},j_{3},j_{4})$, the term corresponds to J is $C(F_{1}{\left(5\right)}^{j_{1}},F_{2}{\left(4\right)}^{j_{2}},F_{3}{\left(1\right)}^{j_{3}}F_{3}{\left(2\right)}^{1-j_{3}},F_{4}{\left(1\right)}^{j_{4}}F_{4}{\left(2\right)}^{1-j_{4}})$.

**Case 5**: $\chi =(x_{1},\cdots ,x_{n})$, $\chi^{\prime }=({x^{\prime }}_{1},\cdots ,{x^{\prime }}_{n})$, where $x_{i_{j}}={x^{\prime }}_{i_{j}}=N+1(j=1,\cdots ,m),$ $x_{i_{j}}\in \{1,\cdots ,N\}(j=m+1,\cdots ,n),{x^{\prime }}_{i_{l}}=N+1(l=m+1,\cdots ,h)$ $x_{i_{s}},{x^{\prime }}_{i_{s}}\in \{1,\cdots ,N\}$ and $x_{i_{s}}\leq {x^{\prime }}_{i_{s}}$ (s=h+1,⋯,n).

Under case 5, at the inspection epoch (4K+l−1delta, component $i_{j}(j=1,\cdots ,m)$ fails, the other components are in working states, at the inspection epoch (4K+ldelta, component $i_{l}(l=m+1,\cdots ,h)$ fails and the remaining n−h components are still in working state. Using the similar methods to those of cases 1–4, the transition probability under case 5 can be given as follows,


$$\begin{array}{cc} & R(\chi^{\prime }|\chi )=\sum_{J\in {\left\{0,1\right\}}^{n-m}}{\left(-1\right)}^{\sum_{k=m+1}^{n}j_{k}}C_{S_{i_{m}}⋃S_{i_{h-m}}⋃\overset{―}{S_{i_{m}}⋃S_{i_{h-m}}}}(1,\cdots ,1,F_{i_{m+1}}{\left(N+1-x_{i_{m+1}}\right)}^{j_{m+1}},\cdots ,\\ & \;F_{i_{h}}{\left(N+1-x_{i_{h}}\right)}^{j_{h}},{\left[F_{i_{h+1}}(1)\right]}^{1_{\left\{{x^{\prime }}_{i_{h+1}}-x_{i_{h+1}}=0\right\}}}{\left[F_{i_{h+1}}{\left({x^{\prime }}_{i_{h+1}}-x_{i_{h+1}}\right)}^{j_{h+1}}F_{i_{h+1}}{\left({x^{\prime }}_{i_{h+1}}-x_{i_{h+1}}+1\right)}^{1-j_{h+1}}\right]}^{1_{\left\{{x^{\prime }}_{i_{h+1}}-x_{i_{h+1}}\neq 0\right\}}},\\ & \;\cdots ,{\left[F_{i_{n}}(1)\right]}^{1_{\left\{{x^{\prime }}_{i_{n}}-x_{i_{n}}=0\right\}}}{\left[F_{i_{n}}{\left({x^{\prime }}_{i_{n}}-x_{i_{n}}\right)}^{j_{n}}F_{i_{n}}{\left({x^{\prime }}_{i_{n}}-x_{i_{n}}+1\right)}^{1-j_{n}}\right]}^{1_{\left\{{x^{\prime }}_{i_{n}}-x_{i_{n}}\neq 0\right\}}}.\; \end{array}$$ (9)

If N+1=6, then χ=(6,2,3,4), $\chi^{\prime }=(6,\;6,\;4,\;5)$ meet with the conditions of case 5. The states of components 1 and 2 are the same within δ time interval. According to Equation 8, there are 8 terms on the right side of it. For $J=(j_{1},j_{2},j_{3})$, the term corresponds to J is $C(1,F_{2}{\left(4\right)}^{j_{1}},F_{3}{\left(1\right)}^{j_{2}}F_{3}{\left(2\right)}^{1-j_{2}},F_{4}{\left(1\right)}^{j_{3}}F_{4}{\left(2\right)}^{1-j_{3}})$.

## 5. Four-stage MDP model

In this section we formulate the alternate replacement problem within the framework of a four-stage MDP. The algorithm to get the discretized state transition probability matrix and backward dynamic programming algorithm are designed to get the optimal alternate replacement strategy.

### 5.1. The value functions

Given the system state $\chi_{l}$ (l=1,2,3,4) at inspection epoch 4Kδ+(l−1delta, let $V_{l}(\chi_{l})$ be the minimal cost over [4Kδ+(l−1delta,4Kδ+4δ). Under the MDP structure, $V_{l}(\chi_{l})(l=1,2,3,4)$ is the value function and can be determined by Bellman equations. For simplicity, we denote the inspection epoch 4Kδ as decision epoch 1, the inspection epoch 4Kδ+δ as decision epoch 2, the inspection epoch 4Kδ+2δ as decision epoch 3, the inspection epoch 4Kδ+3δ as decision epoch 4.

Let $\chi_{1}=(x_{1_{1}},\cdots ,x_{1_{n}})$ be the system state at decision epoch 1. According the modeling assumptions, when the system fails at decision epoch 1, i. e., $\sum_{i=1}^{n}1_{\left\{x_{1_{i}}=N+1\right\}}\geq k$, the system will be replaced in entirety that incurs a set-up cost $C_{s}$, the replacement cost $C_{r}(\chi_{1},a_{1})$ and $C_{r}(\chi_{1},a_{1})$. In the case that the system still survives upon the inspection, we can chose the action from ${𝒜}_{s}=\{a_{1},a_{0}\}$ that minimizes the future expected cost. For action $a_{1}$, the cost at the first stage $r_{1}(\chi_{1},a_{1})$ includes three parts: the cost for replacement in entirety $C_{r}(\chi_{1},a_{1})$, the set-up cost after the replacement $C_{s}$, the failure penalty cost over (4Kδ,4Kδ+δ) $C_{pc}(M^{a_{1}}(\chi_{1}))$. After the replacement in entirety, the number of failed components is zero, hence $C_{pc}(M^{a_{1}}(\chi_{1}))=C_{f}g(0)$. For action $a_{0}$, no maintenance action is taken, hence the cost over the first stage $r_{1}(\chi_{1},a_{0})$ is equal to the failure penalty cost $C_{pc}(M^{a_{0}}(\chi_{1}))$ and $C_{pc}(M^{a_{0}}(\chi_{1}))=C_{f}g(\frac{l_{\chi_{1}}}{n})$, where $l_{\chi_{1}}=\sum_{i=1}^{n}1_{\left\{x_{1_{i}}=N+1\right\}}$. Therefore, $V_{1}(\chi_{1})$ can be given in a form of the following Bellman equations


$$V_{1}(\chi_{1})=\left\{ \begin{array}{c} {}*35lC_{r}(\chi_{1},a_{1})+C_{s}+C_{pc}(M^{a_{1}}(\chi_{1}))+U_{1}^{a_{1}}(\chi_{1}),\;\;\sum_{i=1}^{n}1_{\left\{x_{1_{i}}=N+1\right\}}\geq k,\\ \min\{C_{pc}(M^{a^{0}}(\chi_{1}))+U_{1}^{a^{0}}(\chi_{1}),\\ \;C_{r}(\chi_{1},a_{1})+C_{s}+C_{pc}(M^{a_{1}}(\chi_{1}))+U_{1}^{a_{1}}(\chi_{1})\},\sum_{i=1}^{n}1_{\left\{x_{1_{i}}=N+1\right\}}<k, \end{array}\right.$$ (10)

where $U_{1}^{a_{1}}(\chi_{1})=E[V_{2}|X_{4K\delta }^{+}=\chi^{0}]$, $U_{1}^{a_{0}}(\chi_{1})=E[V_{2}|X_{4K\delta }^{+}=\chi_{1}]$ and they are the expected value functions at the next inspection epoch (the decision epoch 2) given the system states after the maintenance actions. $U_{1}^{a_{1}}(\chi_{1})$ and $U_{1}^{a_{0}}(\chi_{1})$ are given by


$$U_{1}^{a_{1}}(\chi_{1})=\sum \chi \in S^{n}R(\chi |\chi^{0})\;V_{2}(\chi ),$$ (11)

$$U_{1}^{a_{0}}(\chi_{1})=\sum \chi \in S^{n}R(\chi |\chi_{1})\;V_{2}(\chi ).$$ (12)

At the decision epoch 2, when the system fails, the system will also be replaced in entirety. Otherwise, an action will be chosen from ${𝒜}_{u}=\{a|a=(a_{1},\cdots ,a_{n}),\;$ $a_{i}=\{0,1\},i=1,\cdots ,n\}$ such that the future expected cost will be minimized. Given an action a and a system state $\chi_{2}$, the cost over the second stage $r_{2}(\chi_{2},a)$ is the sum of the cost for the replacements in component level $C_{r}(\chi_{2},a)$, the system set-up cost $C_{s}(a)$ and the failure penalty cost over (4Kδ+δ,4Kδ+2δ), denoted by $C_{pc}(M^{a}(\chi_{2}))$. Furthermore, if component i(i=1,⋯,n) is replaced, a cost $c_{i}$ will occur, hence $C_{r}(\chi_{2},a)=\sum_{i=1}^{n}c_{i}1_{\left\{a_{i}=1\right\}}$. The set-up cost $c_{s}$ will occur if at least one component fails, that is at least $a_{i}=1$ (i=1,⋯,n), consequently


$$C_{s}(a)=c_{s}(1-\prod_{i=1}^{n}\left(1-a_{i}\right)).$$

To sum up,


$$V_{2}(\chi_{2})=\left\{ \begin{array}{c} {}*35lC_{r}(\chi_{2},a_{1})+C_{s}(a_{1})+C_{pc}(Ma_{1}(\chi_{2}))+U_{2}^{a_{1}}(\chi_{2}),\;\sum_{i=1}^{n}1_{\left\{x_{2_{i}}=N+1\right\}}\geq k,\\ \min a\in 𝒜u\;\{C_{r}(\chi_{2},a)+C_{s}(a)+C_{pc}(M^{a}(\chi_{2}))+U_{2}^{a}(\chi_{2})\},\;\sum_{i=1}^{n}1_{\left\{x_{2_{i}}=N+1\right\}}<k, \end{array}\right.$$ (13)

where $U_{2}^{a}(\chi_{2})=E[V_{3}|X_{4K\delta +\delta }^{+}=M^{a}(\chi_{2})]$ is the expected value function at the next decision epoch (the decision epoch 3), given the system state after the action a, it can be expressed as


$$U_{2}^{a}(\chi_{2})=\sum \chi \in S^{n}R(\chi |M^{a}(\chi_{2}))\;V_{3}(\chi ).$$ (14)

By the similar method to that of $V_{1}(\chi_{1})$, for the system state $\chi_{3}=(x_{3_{1}},\cdots ,x_{3_{n}})$ at the inspection epoch 4Kδ+2δ, the value function can be given by the following Bellman equations


$$V_{3}(\chi_{3})=\left\{ \begin{array}{c} {}*35lC_{r}(\chi_{3},a_{1})+C_{s}+C_{pc}(M^{a_{1}}(\chi_{3}))+U_{3}^{a_{1}}(\chi_{3}),\;\;\sum_{i=1}^{n}1_{\left\{x_{3_{i}}=N+1\right\}}\geq k,\\ \min\{C_{pc}(M^{a_{0}}(\chi_{3}))+U_{3}^{a_{0}}(\chi_{3}),\\ \;C_{r}(\chi_{3},a_{1})+C_{s}+C_{pc}(M^{a_{1}}(\chi_{3}))+U_{3}^{a_{1}}(\chi_{3})\},\;\sum_{i=1}^{n}1_{\left\{x_{3_{i}}=N+1\right\}}<k. \end{array}\right.$$ (15)

By the similar method to that of $V_{2}(\chi_{2})$, for the system state $\chi_{4}=(x_{4_{1}},\cdots ,x_{4_{n}})$ at the inspection epoch 4Kδ+3δ, $V_{4}(\chi_{4})$ can be given by the following Bellman equations


$$V_{4}(\chi_{4})=\left\{ \begin{array}{c} {}*35lC_{r}(\chi_{4},a_{1})+C_{s}+C_{pc}(M^{a_{1}}(\chi_{4}))+U_{4}^{a_{1}}(\chi_{4}),\;\sum_{i=1}^{n}1_{\left\{x_{4_{i}}=N+1\right\}}\geq k,\\ \min a\in 𝒜u\;\{C_{r}(\chi_{4},a)+C_{s}+C_{pc}(M^{a}(\chi_{4}))+U_{4}^{a}(\chi_{4})\},\;\sum_{i=1}^{n}1_{\left\{x_{4_{i}}=N+1\right\}}<k, \end{array}\right.$$ (16)

where $U_{4}^{a_{1}}(\chi_{4})=E[V_{5}|X_{4K\delta +3\delta }^{+}=\chi^{0}]$ and $U_{4}^{a}(\chi_{4})=E[V_{5}|X_{4K\delta +3\delta }^{+}=M^{a}(\chi_{4})]$ are expected value functions at next decision epoch (the decision epoch 5), given the system states after the actions $a_{1}$ and a, respectively. They can be obtained by the following expressions


$$\begin{array}{c} U_{4}^{a_{1}}(\chi_{4})=\sum \chi \in S^{n}R(\chi |\chi^{0})\;V_{5}(\chi ),\;\;U_{4}^{a}(\chi_{4})=\sum \chi \in S^{n}R(\chi |M^{a}(\chi_{4}))\;V_{5}(\chi ) \end{array}$$

where $V_{1}(\chi_{1})$, χ is the salvage value of the system corresponding to the state χ in the end of the fourth stage, γ(0<γ<1) is the depreciation coefficient, r is a decreasing function of the failure component number $l_{\chi }$.

**Fig 4:**
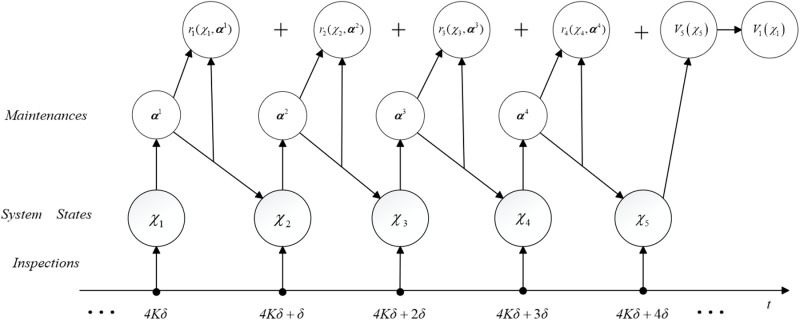
The maintenance decision-making process for the railway grid.

The maintenance decision process of the four-stage MDP is sketched in Fig 4. The maintenance actions $a^{1}$, $a^{3}$ and $a^{2}$, $a^{4}$ are chosen from state spaces ${𝒜}_{s}$ and ${𝒜}_{u}$, respectively, such that the total expected cost over one year $V_{1}(\chi_{1})$ is minimized.

### 5.2. Algorithm to obtain state transition probability matrix

For the convenience of our statement, the states before and after a transition will be numbered. We denote the state order number before a transition by h and that after a transition by $h^{\prime }$. In the following, i is the component order number. $R_{hh^{\prime }}$ is the h th row and the $h^{\prime }$ th column element of matrix R. $J_{l}$ is a vector of n-dimension whose elements are 0 or 1. According the results in Section 3.2, the following algorithm is proposed to calculate the state transition probability matrix.

**Table:** 

Algorithm 1: State Transition Probability Matrix Algorithm
**Input:** The distribution functions of degradation increment over δ : $F_{i}(x_{i})$ ; Copula function: C . Discretization level: Δd . The number of component state: N+1 _._ **Output:** State transition probability matrix R .
Initialization R=0. Generate system state spaces before and after a transition $\chi =\{\chi^{h}|\chi^{h}=(x_{1}^{h},\cdots ,x_{n}^{h}\}$ and $\chi^{\prime }=\{\chi^{h^{\prime }}|\chi^{h^{\prime }}=(x_{1}^{h^{\prime }},\cdots ,x_{n}^{h^{\prime }}\}$_._ For $h=1:{\left(N+1\right)}^{n}$ and $h^{\prime }=1:{\left(N+1\right)}^{n}$, assignment values to degradation increment distributions according to the results in Section 3.2 as follows. for i=1:n If $x_{i}^{h}>x_{i}^{h^{\prime }}$, $R_{hh^{\prime }}=0$, go to step 5. If $x_{i}^{h},x_{i}^{h^{\prime }}\in \{1,\cdots ,N\}$, calculate $F_{i}((x_{i}^{h^{\prime }}-x_{i}^{h}Deltad)$ and $F_{i}((x_{i}^{h^{\prime }}-x_{i}^{h}+1Deltad)$, $F_{i}(x_{i}leftarrowF_{i}((x_{i}^{h^{\prime }}-x_{i}^{h})\Delta d)$, $F_{i}(x_{i}+1leftarrowF_{i}((x_{i}^{h^{\prime }}-x_{i}^{h}+1)\Delta d)$, i←i+1; If $x_{i}^{h}\neq N+1,x_{i}^{h^{\prime }}=N+1$, calculate $F_{i}((x_{i}^{h^{\prime }}-x_{i}^{h}Deltad)$, $F_{i}(x_{i}^{h^{\prime }}-x_{i}^{h}+1leftarrow1$, $F_{i}(x_{i}leftarrowF_{i}((x_{i}^{h^{\prime }}-x_{i}^{h})\Delta d)$, i←i+1; If $x_{i}^{h}=N+1$, $F_{i}((x_{i}^{h^{\prime }}-x_{i}^{h})leftarrow1$, $F_{i}((x_{i}^{h^{\prime }}-x_{i}^{h}+1)leftarrow1$, i←i+1. end Calculate $R_{hh^{\prime }}$ as follows. for $l=1:2^{n}$ Generate $J_{l}=(j_{l_{1}},\cdots ,j_{l_{n}})$; Calculate the value of copula function C in equations of Section 4.2 and assign it to c; Calculate $Sign(J_{l})$ according to the results in Section 4.2; $R_{hh^{\prime }}\leftarrow R_{hh^{\prime }}+c\times {\left(-1\right)}^{sign(J_{l})}$, l←l+1. end Output $R_{hh^{\prime }}$, h←h+1, $h^{\prime }\leftarrow h^{\prime }+1$, go to step 3. Output the transition probability matrix R.

### 5.3. Backward dynamic programming algorithm

To obtain the optimal alternate replacement policy of system level and component level, a backward dynamic programming algorithm will be given in this section. In the algorithm, t is the order number of stages, j is the order number of actions, |𝒜| is the number of elements in set 𝒜, mincosts is the temporary variable for the value functions, minaction is temporary variable for the optimal action. The detail of the algorithm is as follows.

**Table:** 

Algorithm 2: Backward Dynamic Programming Algorithm Under Four Stages
**Input:** K , $c_{i},\;i=1,\cdots n$ , $C_{r}(\chi ,a)$ , $C_{s}(a)$ , $M^{a}(\chi )$ , $C_{pc}(\chi )$ , $V_{5}(\chi )$ **Output:** The optimal alternate replacement strategy and values functions
Initialization: t=5, j=0, c=0, mincosts=+∞, $minaction=a_{0}$. for t=4:1, generate system state pace $\chi =\{\chi_{t}^{h}=(x_{1}^{h},\cdots ,x_{n}^{h}\}$ at stage t. Generate the action space ${𝒜}_{t}$ at stage t. for $h=1:{\left(N+1\right)}^{n}$, $\chi_{t}^{h}=(x_{1}^{h},\cdots ,x_{n}^{h})$, mincost=+∞, compute $V_{t}(\chi_{t}^{h})$. Computer the number of failure component. If the number is equal to or larger than k, c←0, $c\leftarrow C_{r}(\chi^{k},a_{1})+C_{s}(a)+C_{pc}(\chi^{0}))$. Calculate $U_{t}^{a_{1}}(\chi_{t}^{h})$ by algorithm 1 and the values function of stage t+1, $V_{t}(\chi_{t}^{h}leftarrowc+U_{t}^{a_{1}}(\chi_{t}^{h})$, $minaction\leftarrow a_{1}$. Go to step 5. If the number is smaller than k, go to step 4. for $j=1:|𝒜{|}_{t}$ c←0, $c\leftarrow C_{r}(\chi_{t}^{h},a_{j})+C_{s}(a_{j})+C_{pc}(M^{a_{j}}(\chi_{t}^{h}))$. Calculate $U_{t}^{a_{j}}(\chi_{t}^{k})$ by Algorithm 1 and the values function of stage t+1 $c\leftarrow c+U_{t}^{a_{j}}(\chi_{t}^{k})$. If c<mincost, mincost←c, $minaction\leftarrow a_{j}$. end $V_{t}(\chi_{t}^{h}leftarrowmincost$. Output $V_{t}(\chi_{t}^{h})$ and minaction, h←h+1, go to step 3. end Output the value functions of and optimal policies for stage t, t←t−1, go to Step 2. Output the value functions of and optimal policies for four stages.

### 5.4. Properties of the value functions

In this section, the monotonicity of value functions will be discussed [38–40]. Before presenting the main results, some definitions and a theorem on stochastic orders are listed firstly in the following.

**Definition 1** For two system states $\chi =(x_{1},\cdots ,x_{n})$, $\chi^{\prime }=({x^{\prime }}_{1},\cdots ,{x^{\prime }}_{n})$, we denote $\chi <\chi^{\prime }$ if and only if $x_{i}<{x^{\prime }}_{i}(i=1,\cdots ,n)$ [41].

Obviously, if $\chi <\chi^{\prime }$, then the degradation states of all components for state χ are

worse than those of state $\chi^{\prime }$. The order is a partial one.

**Definition 2** Let ϕ be a univariate or a multivariate function with domain in ${\mathbb{R}}^{n}$. If $\phi (\chi le\phi (\chi^{\prime })$, whenever $\chi <\chi^{\prime }$, then we say that the function ϕ is increasing [41].

**Definition 3** Let X and Y be two random vectors such that

P{X∈U}≤P{Y∈U} for all upper sets $U\subset {\mathbb{R}}^{n}$.

Then X is said to be smaller than Y in the usual stochastic order (denoted by $X_{st}\leq Y$).

$X_{st}\leq Y$ if, and only if, E[ϕ(X)]≤E[ϕ(Y)] holds for all increasing functions ϕ for which the expectations exist [41].

**Theorem 1** Let the random vector X and Y have a common copula and $X=(X_{1},X_{2},\cdots ,X_{n})$, $Y=(Y_{1},Y_{2},\cdots ,Y_{n})$. If $X_{i}\leq_{st}Y_{i}\;,\;\;i=1,\;2,\;\cdots ,n$, then $X_{st}\leq Y$ [41].

The result of Theorem 1 is significant because it allows the traditional stochastic order problem involving random vectors to be transformed into a simpler problem of univariate random variables, which is more straightforward to verify, provided that they share a common copula. To explore the monotonicity of the value functions presented in this paper, we first outline several key propositions.

**Proposition 1** For a system state $\chi =(x_{1},\cdots ,x_{n})$, the number of failed components $l_{\chi }$ is increasing in χ.

**Proof:** Consider two system states $\chi =(x_{1},\cdots ,x_{n})$, $\chi^{\prime }=({x^{\prime }}_{1},\cdots ,{x^{\prime }}_{n})$ such that

$\chi <\chi^{\prime }$. If $x_{i}<{x^{\prime }}_{i}<N+1$, then $1_{\left\{x_{i}=N+1\right\}}=1_{\left\{{x^{\prime }}_{i}=N+1\right\}}=0$. If $x_{i}<N+1,{x^{\prime }}_{i}=N+1$, then $1_{\left\{x_{i}=N+1\right\}}<1_{\left\{{x^{\prime }}_{i}=N+1\right\}}$. If $x_{i}={x^{\prime }}_{i}=N+1$, then $1_{\left\{x_{i}=N+1\right\}}=1_{\left\{{x^{\prime }}_{i}=N+1\right\}}=1$. To sum up, if $\chi <\chi^{\prime }$, then $1_{\left\{x_{i}=N+1\right\}}\leq 1_{\left\{{x^{\prime }}_{i}=N+1\right\}}$ (i=1,⋯,n). Furthermore, $l_{\chi }=\sum_{i=1}^{n}1_{\left\{x_{i}=N+1\right\}}$, $l_{\chi^{\prime }}=\sum_{i=1}^{n}1_{\left\{{x^{\prime }}_{i}=N+1\right\}}$. Hence $l_{\chi }$ is increasing as χ increases. ■

According to Proposition 1, as the condition of a system worsens, the number of failed components is likely to increase. This suggests a direct correlation between the deteriorating state of the system and the frequency of component failures. In essence, as the overall integrity of the system declines, the probability of experiencing additional failures rises, highlighting the importance of maintaining system health to prevent a cascade of breakdowns.

**Proposition 2** For a maintenance action $a=(a_{1},\cdots ,a_{n})$, the system state after a, $M^{a}(\chi )$ is increasing as χ increases.

**Proof:** Let $\chi =(x_{1},\cdots ,x_{n})$ and $\chi^{\prime }=({x^{\prime }}_{1},\cdots ,{x^{\prime }}_{n})$ are two system states such that $\chi <\chi^{\prime }$. For a maintenance action $a=(a_{1},\cdots ,a_{n})$, if $a_{i}=0$, $x_{i}^{1-a_{i}}=x_{i},$ ${x_{i}^{\prime }}^{1-a_{i}}={x^{\prime }}_{i}$, hence $x_{i}^{1-a_{i}}<{x_{i}^{\prime }}^{1-a_{i}}$. For $a_{i}=1$, $x_{i}^{1-a_{i}}={x_{i}^{\prime }}^{1-a_{i}}=1$. Thus, if $\chi <\chi^{\prime }$, $x_{i}^{1-a_{i}}\leq {x_{i}^{\prime }}^{1-a_{i}}$. From definition 1, $M^{a}(\chi leM^{a}(\chi^{\prime })$ ■.

Proposition 2 suggests that a deteriorated initial state corresponds to a worse the condition of the system. By the increasing property of the function g and Propositions 1 and 2, we can obtain the following corollary.

**Corollary 1** For any maintenance action $a=(a_{1},\cdots ,a_{n})$, $C_{pc}(M^{a}(\chi ))$ is increasing in χ.

Corollary 1 indicates that the expected penalty cost of system failure over an inspection interval increases as the initial state increases. On the monotonicity of value functions, we have the following theorem.

**Theorem 2** The value function $V_{t}(\chi_{t},(t=1,2,3,4,5)$ is increasing as $\chi_{t}$ increases.

**Proof:** We prove Theorem 2 via the mathematical induction method. Recall that $V_{5}(\chi_{5})$, γ(0<γ<1) and r is a decreasing function of the failed component number $l_{\chi_{5}}$, $V_{5}(\chi_{5})$ is obviously increasing in $l_{\chi_{5}}$. From proposition 2, $l_{\chi_{5}}$ is also increasing in $\chi_{5}$. Thus $V_{5}(\chi_{5})$ is increasing as $\chi_{5}$ increases.

Suppose that, for 1<t<4, $V_{t+1}(\chi_{t+1},$ is increasing as $\chi_{t+1}$ increases, we will show that $V_{t}(\chi_{t},$ is increasing in $\chi_{t}$.

Let $X_{t}^{+}$ denote the system state at stage t after a maintenance action. For two sample of $X_{t}^{+}$, $\chi_{t}=(x_{1},\cdots ,x_{n})$, ${\chi^{\prime }}_{t}=({x^{\prime }}_{1},\cdots ,{x^{\prime }}_{n})$ such that $\chi_{i}<{\chi^{\prime }}_{i}$, we consider the usual stochastic order of the system states $Xt+1|X_{t}^{+}=\chi_{t}$ and $Xt+1|X_{t}^{+}={\chi^{\prime }}_{t}$.

$Xt+1|X_{t}^{+}=\chi_{t}$ and $X_{t+1}^{+}|X_{t}^{+}={\chi^{\prime }}_{t}$ are the system state increments over time interval δ giver the initial values ${\chi^{\prime }}_{t}$ and ${\chi^{\prime }}_{t}$, consequently they have the common copula function C. Suppose that Xi (i=1,2,⋯,n) is the degradation level of component i at stage t+1, given the state at stage t after a maintenance action, that is $Xi=Xt+1i|X_{t}^{i+}=x_{i}$. Similarly, let $Yi=X_{t+1}^{i}|X_{t}^{i+}={x^{\prime }}_{i}$ (i=1,2,⋯,n). For each $x\in R^{+},x\geq {x^{\prime }}_{i}$,


$$P\{Xi\leq x\}=P\{X_{\delta }^{i}\leq x-x_{i}\}=F_{i}(x-x_{i})(i=1,\cdots ,n),$$

$$P\{Yi\leq x\}=P\{X_{\delta }^{i}\leq x-{x^{\prime }}_{i}\}=F_{i}(x-{x^{\prime }}_{i})(i=1,\cdots ,n).$$

Due to the nondecreasing property of $F_{i}$, P{Xi≤x}≥P{Yi≤x}. Obviously, for the other $x\in R^{+}$, P{Xi≤x}≥P{Yi≤x} also holds. Hence $X_{i}\leq_{st}Y_{i}\;,\;\;i=1,\;2,\;\cdots ,n$. From Theorem 1, $Xt+1|X_{t}^{+}=\chi_{t}\leq_{st}Xt+1|X_{t}^{+}={\chi^{\prime }}_{t}$. That is $Xt+1|X_{t}^{+}=\chi_{t}$ is increasing in $\chi_{t}$.

According to the increasing property of $V_{t+1}(\chi_{t+1},$ on $\chi_{t+1}$, Proposition 2 and Theorem 1, for a maintenance action a, $U_{t}^{a}(\chi_{t})=E[V_{t+1}|X_{t}^{+}=M^{a}(\chi_{t})]$ is increasing in $\chi_{t}$. Furthermore, in Bellman Equations 10,13,15,16 of section 4.1, $C_{r}(\chi ,a)$ and $C_{s}(a)$ are independent of $\chi_{t}$ and $C_{pc}(M^{a}(\chi_{t}))$ is also increasing in $\chi_{t}$. Hence, $V_{t}(\chi_{t},$ is increasing as $\chi_{t}$ increases. ■

The theorem guarantees that a worse system state will lead to a higher cost to go, which enables decision makers to compare and estimate the future costs based on the current observations.

### 5.5. Comparison models

To illustrate the effectiveness of the alternate replacement strategy with system level and component level proposed in our paper, two comparison models are built in this section.

For comparison model 1, at decision epochs 4Kδ and 4Kδ+2δ, a system replacement will be adopted if the system fails (the number of failed components exceeds the threshold k); Otherwise, nothing is done. At decision epochs 4Kδ+δ and 4Kδ+3δ, the replacement strategy of component level is the same to that of the original model. Let

${V_{l}}^{\prime }(\chi_{l}(l=1,2,3,4)$ be the minimal cost over [4Kδ+(l−1delta,4Kδ+4δ) for comparison model 1. According the model assumptions, at the decision epoch 4Kδ, a system replacement will be adopted if the system fails, hence


$${V^{\prime }}_{1}(\chi_{1})=\left\{ \begin{array}{c} {}*35lC_{r}(\chi_{1},a_{1})+C_{s}(a_{1})+C_{pc}(M^{a_{1}}(\chi_{1}))+U_{1}^{a_{1}}(\chi_{1}),\sum_{i=1}^{n}1_{\left\{x_{1_{i}}=N+1\right\}}\geq k,\\ C_{pc}(M^{a_{0}}(\chi_{1}))+U_{1}^{a_{0}}(\chi_{1}),\;\sum_{i=1}^{n}1_{\left\{x_{1_{i}}=N+1\right\}}<k. \end{array}\right.$$

At the decision epoch 4Kδ+δ, the replacement strategy of component level will be performed, therefore


$${V^{\prime }}_{2}(\chi_{2})=\left\{ \begin{array}{c} {}*35lC_{r}(\chi_{2},a_{1})+C_{s}(a_{1})+C_{pc}(M^{a_{1}}(\chi_{2}))+U_{2}^{a_{1}}(\chi_{2}),\sum_{i=1}^{n}1_{\left\{x_{2_{i}}=N+1\right\}}\geq k,\\ \underset{a\in {𝒜}_{u}}{\min}\;\{C_{r}(\chi_{2},a)+C_{s}(a)+C_{pc}(M^{a}(\chi_{2}))+U_{2}^{a}(\chi_{2})\},\sum_{i=1}^{n}1_{\left\{x_{2_{i}}=N+1\right\}}<k. \end{array}\right.$$

Similarly, ${V^{\prime }}_{3}(\chi_{3})$ and ${V^{\prime }}_{4}(\chi_{4})$ can be given by the following equations


$${V^{\prime }}_{3}(\chi_{3})=\left\{ \begin{array}{c} {}*35lC_{r}(\chi_{3},a_{1})+C_{s}(a_{1})+C_{pc}(M^{a_{1}}(\chi_{3}))+U_{3}^{a_{1}}(\chi_{3}),\sum_{i=1}^{n}1_{\left\{x_{3_{i}}=N+1\right\}}\geq k,\\ C_{pc}(M^{a_{0}}(\chi_{3}))+U_{1}^{a_{0}}(\chi_{3}),\;\sum_{i=1}^{n}1_{\left\{x_{3_{i}}=N+1\right\}}<k. \end{array}\right.$$

$${V^{\prime }}_{4}(\chi_{4})=\left\{ \begin{array}{c} {}*35lC_{r}(\chi_{4},a_{1})+C_{s}(a_{1})+C_{pc}(M^{a_{1}}(\chi_{4}))+U_{4}^{a_{1}}(\chi_{4}),\sum_{i=1}^{n}1_{\left\{x_{4_{i}}=N+1\right\}}\geq k,\\ \underset{a\in {𝒜}_{u}}{\min}\;\{C_{r}(\chi_{4},a)+C_{s}(a)+C_{pc}(M^{a}(\chi_{4}))+U_{2}^{a}(\chi_{4})\},\sum_{i=1}^{n}1_{\left\{x_{4_{i}}=N+1\right\}}<k. \end{array}\right.$$

For comparison model 2, at all decision epochs, the system will be replaced entirely if it fails; Otherwise, nothing is done. Let ${V_{l}}^{\prime \prime }(\chi_{l}(l=1,2,3,4)$ be the minimal cost over [4Kδ+(l−1delta,4Kδ+4δ) for comparison model 2, then they can be obtained recursively by the following equation


$${V_{l}}^{\prime \prime }(\chi_{l})=\left\{ \begin{array}{c} {}*35lC_{r}(\chi_{l},a_{1})+C_{s}(a_{1})+C_{pc}(M^{a_{1}}(\chi_{l}))+\sum \chi_{l+1}\in S^{n}R(\chi_{l+1}|\chi^{0}){V_{l}}^{\prime \prime }(\chi_{l}),\;\sum_{i=1}^{n}1_{\left\{x_{l_{i}}=N+1\right\}}\geq k,\\ C_{pc}(M^{a_{0}}(\chi_{l}))++\sum \chi_{l+1}\in S^{n}R(\chi_{l+1}|\chi_{l+1}){V^{\prime \prime }}_{l+1}(\chi_{l})\;,\;\sum_{i=1}^{n}1_{\left\{x_{l_{i}}=N+1\right\}}<k, \end{array}\right.$$

where


$${V_{5}}^{\prime \prime }(\chi_{5})=V_{5}(\chi_{5}).$$

In comparison model 1, the replacement actions of component level is optimized. In comparison model 2, replacement actions are trigged by system failures. While under the alternate replacement strategy with system level and component level proposed in our paper, the replacement actions of system level and component level are optimized jointly.

## 6. Numerical studies and analysis

In this section, a numerical example will be given to illustrate the feasibility of the model. Sensitivity analysis of dependence strength and cost-related parameters is also performed.

### 6.1. Optimal replacement policy

Consider a 3-out-of 4: F system, the parameters of the Gamma degradation increment processes within δ time interval associated component i (i=1,2,3,4) are $\left(\alpha_{i},\beta_{i}\right)$. They are listed in Table 1.

**Table 1:** Parameters of the Gamma increment processes.

Component 1	Component 2	Component 3	Component 4
$\alpha_{1}=1/8$	$\alpha_{2}=1/11$	$\alpha_{3}=120/801$	$\alpha_{4}=150/1200$
$\beta_{1}=1/7$	$\beta_{2}=1/6$	$\beta_{3}=120/700$	$\beta_{4}=150/600$

Suppose that the dependent structure among the degradation increment processes within δ time interval can be modeled by a Clayton-Copula function as follows


$$\begin{array}{cc} & \;P(X_{\delta }^{1}\leq x_{1}\Delta d,X_{\delta }^{2}\leq x_{2}\Delta d,X_{\delta }^{3}\leq x_{3}\Delta d,X_{\delta }^{4}\leq x_{4}\Delta d)\\ & =C(F_{1}(x_{1}),F_{2}(x_{2}),F_{3}(x_{3}),F_{4}(x_{4}))\\ & ={\left(F_{1}{\left(x_{1}\right)}^{-\theta }+F_{2}{\left(x_{2}\right)}^{-\theta }+F_{3}{\left(x_{3}\right)}^{-\theta }+F_{4}{\left(x_{4}\right)}^{-\theta }-3\right)}^{-\;\frac{1}{\theta }}, \end{array}$$

where Δd=5, θ=2, δ=3.

The degradation states of the four components are discretized into three states, named 1, 2, 3. The failure threshold L=2Δd. The costs parameters are shown in Table 2.

**Table 2:** Cost parameters.

$c_{1}$	$c_{2}$	$c_{3}$	$c_{4}$	$C_{s}$	$C_{f}$	γ
40	90	50	100	110	50	0.8

The penalty cost of system failures over an inspection interval $C_{pc}(\chi )=C_{f}e^{\frac{l_{\chi }}{n}}$. The value function for stage 5 is $V_{5}(\chi )=-\gamma \frac{l_{\chi }}{n}\sum_{i=1}^{4}c_{i}$.

By Algorithm 1 and Matlab software we can calculate the state transition probability matrix R. The value functions and the associated alternate replacement strategy can also be obtained by using Algorithm 2. The value functions and the associated alternate replacement strategies under stages 1 and 2 are as shown in Tables 3 and 4.

**Table 3:** The value functions and optimal strategy for stage 1.

States	Actions	$V_{1}$	States	Actions	$V_{1}$
(1,1,1,1)	(0,0,0,0)	504.72	(2,1,1,1)	(0,0,0,0)	535.88
(1,1,1,2)	(0,0,0,0)	535.59	(2,1,1,2)	(0,0,0,0)	572.87
(1,1,1,3)	(0,0,0,0)	662.26	(2,1,1,3)	(0,0,0,0)	716.12
(1,1,2,1)	(0,0,0,0)	538.49	(2,1,2,1)	(0,0,0,0)	575.40
(1,1,2,2)	(0,0,0,0)	576.23	(2,1,2,2)	(0,0,0,0)	622.24
(1,1,2,3)	(0,0,0,0)	721.98	(2,1,2,3)	(0,0,0,0)	795.36
(1,1,3,1)	(0,0,0,0)	621.46	(2,1,3,1)	(0,0,0,0)	663.78
(1,1,3,2)	(0,0,0,0)	666.12	(2,1,3,2)	(0,0,0,0)	721.11
(1,1,3,3)	(0,0,0,0)	824.20	(2,1,3,3)	(1,1,1,1)	894.72
(1,2,1,1)	(0,0,0,0)	534.62	(2,2,1,1)	(0,0,0,0)	572.19
(1,2,1,2)	(0,0,0,0)	570.03	(2,2,1,2)	(0,0,0,0)	613.26
(1,2,1,3)	(0,0,0,0)	710.23	(2,2,1,3)	(0,0,0,0)	771.46
(1,2,2,1)	(0,0,0,0)	575.71	(2,2,2,1)	(0,0,0,0)	623.57
(1,2,2,2)	(0,0,0,0)	617.02	(2,2,2,2)	(0,0,0,0)	668.28
(1,2,2,3)	(0,0,0,0)	776.75	(2,2,2,3)	(0,0,0,0)	844.63
(1,2,3,1)	(0,0,0,0)	665.86	(2,2,3,1)	(0,0,0,0)	724.08
(1,2,3,2)	(0,0,0,0)	714.37	(2,2,3,2)	(0,0,0,0)	775.76
(1,2,3,3)	(0,0,0,0)	875.19	(2,2,3,3)	(1,1,1,1)	894.72
(1,3,1,1)	(0,0,0,0)	649.47	(2,3,1,1)	(0,0,0,0)	701.58
(1,3,1,2)	(0,0,0,0)	696.52	(2,3,1,2)	(0,0,0,0)	755.48
(1,3,1,3)	(0,0,0,0)	874.02	(2,3,1,3)	(1,1,1,1)	894.72
(1,3,2,1)	(0,0,0,0)	707.61	(2,3,2,1)	(0,0,0,0)	782.67
(1,3,2,2)	(0,0,0,0)	760.92	(2,3,2,2)	(0,0,0,0)	830.27
(1,3,2,3)	(1,1,1,1)	894.72	(2,3,2,3)	(1,1,1,1)	894.72
(1,3,3,1)	(0,0,0,0)	808.08	(2,3,3,1)	(0,0,0,0)	888.15
(1,3,3,2)	(0,0,0,0)	857.56	(2,3,3,2)	(1,1,1,1)	894.72
(1,3,3,3)	(1,1,1,1)	1374.7	(2,3,3,3)	(1,1,1,1)	1374.7
(3,1,1,1)	(0,0,0,0)	618.37	(3,2,2,3)	(1,1,1,1)	894.72
(3,1,1,2)	(0,0,0,0)	661.77	(3,2,3,1)	(0,0,0,0)	800.29
(3,1,1,3)	(0,0,0,0)	811.59	(3,2,3,2)	(0,0,0,0)	850.35
(3,1,2,1)	(0,0,0,0)	663.67	(3,2,3,3)	(1,1,1,1)	1374.7
(3,1,2,2)	(0,0,0,0)	720.66	(3,3,1,1)	(0,0,0,0)	795.15
(3,1,2,3)	(1,1,1,1)	894.72	(3,3,1,2)	(0,0,0,0)	845.92
(3,1,3,1)	(0,0,0,0)	740.31	(3,3,1,3)	(1,1,1,1)	1374.7
(3,1,3,2)	(0,0,0,0)	796.14	(3,3,2,1)	(0,0,0,0)	881.76
(3,1,3,3)	(1,1,1,1)	1374.7	(3,3,2,2)	(1,1,1,1)	894.72
(3,2,1,1)	(0,0,0,0)	661.22	(3,3,2,3)	(1,1,1,1)	1374.7
(3,2,1,2)	(0,0,0,0)	709.09	(3,3,3,1)	(1,1,1,1)	1374.7
(3,2,1,3)	(0,0,0,0)	863.92	(3,3,3,2)	(1,1,1,1)	1374.7
(3,2,2,1)	(0,0,0,0)	723.61	(3,3,3,3)	(1,1,1,1)	1374.7
(3,2,2,2)	(0,0,0,0)	772.24			

**Table 4:** The value functions and optimal strategy for stage 2.

States	Actions	$V_{2}$	States	Actions	$V_{2}$
(1,1,1,1)	(0,0,0,0)	425.30	(2,1,1,1)	(0,0,0,0)	449.25
(1,1,1,2)	(0,0,0,0)	446.26	(2,1,1,2)	(0,0,0,0)	475.13
(1,1,1,3)	(0,0,0,0)	558.95	(2,1,1,3)	(0,0,0,0)	605.29
(1,1,2,1)	(0,0,0,0)	451.26	(2,1,2,1)	(0,0,0,0)	480.44
(1,1,2,2)	(0,0,0,0)	477.67	(2,1,2,2)	(0,0,0,0)	514.45
(1,1,2,3)	(0,0,0,0)	609.77	(2,1,2,3)	(0,0,0,0)	676.09
(1,1,3,1)	(0,0,0,0)	532.15	(2,1,3,1)	(0,0,0,0)	569.35
(1,1,3,2)	(0,0,0,0)	566.22	(2,1,3,2)	(0,0,0,0)	615.87
(1,1,3,3)	(0,0,1,1)	685.30	(2,1,3,3)	(0,0,1,1)	709.25
(1,2,1,1)	(0,0,0,0)	445.97	(2,2,1,1)	(0,0,0,0)	475.01
(1,2,1,2)	(0,0,0,0)	470.57	(2,2,1,2)	(0,0,0,0)	504.65
(1,2,1,3)	(0,0,0,0)	596.42	(2,2,1,3)	(0,0,0,0)	651.33
(1,2,2,1)	(0,0,0,0)	477.65	(2,2,2,1)	(0,0,0,0)	515.82
(1,2,2,2)	(0,0,0,0)	507.51	(2,2,2,2)	(0,0,0,0)	549.19
(1,2,2,3)	(0,0,0,0)	655.52	(2,2,2,3)	(0,0,0,0)	717.87
(1,2,3,1)	(0,0,0,0)	566.44	(2,2,3,1)	(0,0,0,0)	618.97
(1,2,3,2)	(0,0,0,0)	605.18	(2,2,3,2)	(0,0,0,0)	661.47
(1,2,3,3)	(0,0,1,1)	705.97	(2,2,3,3)	(0,0,1,1)	735.01
(1,3,1,1)	(0,0,0,0)	549.99	(2,3,1,1)	(0,0,0,0)	594.53
(1,3,1,2)	(0,0,0,0)	586.03	(2,3,1,2)	(0,0,0,0)	638.53
(1,3,1,3)	(0,1,0,1)	725.30	(2,3,1,3)	(0,1,0,1)	749.25
(1,3,2,1)	(0,0,0,0)	599.10	(2,3,2,1)	(0,0,0,0)	666.60
(1,3,2,2)	(0,0,0,0)	642.79	(2,3,2,2)	(0,0,0,0)	706.14
(1,3,2,3)	(0,1,0,1)	751.26	(2,3,2,3)	(0,1,0,1)	780.44
(1,3,3,1)	(0,1,1,0)	675.30	(2,3,3,1)	(0,1,1,0)	699.25
(1,3,3,2)	(0,1,1,0)	696.26	(2,3,3,2)	(0,1,1,0)	725.13
(1,3,3,3)	(1,1,1,1)	1295.3	(2,3,3,3)	(1,1,1,1)	1295.3
(3,1,1,1)	(0,0,0,0)	529.71	(3,2,2,3)	(1,0,0,1)	727.65
(3,1,1,2)	(0,0,0,0)	562.95	(3,2,3,1)	(1,0,1,0)	645.97
(3,1,1,3)	(1,0,0,1)	675.30	(3,2,3,2)	(1,0,1,0)	670.57
(3,1,2,1)	(0,0,0,0)	569.45	(3,2,3,3)	(1,1,1,1)	1295.3
(3,1,2,2)	(0,0,0,0)	616.01	(3,3,1,1)	(1,1,0,0)	665.30
(3,1,2,3)	(1,0,0,1)	701.26	(3,3,1,2)	(1,1,0,0)	686.26
(3,1,3,1)	(1,0,1,0)	625.30	(3,3,1,3)	(1,1,1,1)	1295.3
(3,1,3,2)	(1,0,1,0)	646.26	(3,3,2,1)	(1,1,0,0)	691.26
(3,1,3,3)	(1,1,1,1)	1295.3	(3,3,2,2)	(1,1,0,0)	717.67
(3,2,1,1)	(0,0,0,0)	562.97	(3,3,2,3)	(1,1,1,1)	1295.3
(3,2,1,2)	(0,0,0,0)	601.51	(3,3,3,1)	(1,1,1,1)	1295.3
(3,2,1,3)	(1,0,0,1)	695.97	(3,3,3,2)	(1,1,1,1)	1295.3
(3,2,2,1)	(0,0,0,0)	619.11	(3,3,3,3)	(1,1,1,1)	1295.3
(3,2,2,2)	(1,0,0,0)	657.51			

In stage 1 of the maintenance strategy, if the system fails—defined by having at least three failed components, then the entire system will be replaced. Conversely, if the system is in a functioning state, it is permissible either to replace it entirely or to take no action at all. Table 3 illustrates that for eight specific failed states—namely (1,3,3,3), (2,3,3,3), (3,1,3,3), (3,2,3,3), (3,3,2,3), (3,3,3,1), (3,3,3,2), and (3,3,3,3)—the designated action is to replace the system in its entirety, and notably, the value functions for these states are uniform. This indicates a clear decision to replace the system when it reaches a critical level of failure. For other system states, a clear trend emerges: as the severity of the system state worsens, the likelihood of opting for a complete replacement increases. For example, in the first group, action (0,0,0,0)—representing the decision to take no action—remains prevalent, but its frequency declines in subsequent groups [2 and 3]. This decreasing trend aligns with the decision rules established for the model.

In stage 2, the replacement strategy focuses on component-level interventions. Specifically, if the system experiences a failure—defined as having at least three failed components—a complete replacement of the affected system is mandated. Conversely, if the system is not deemed to have failed, individual components may either be replaced or left untouched based on their condition. Table 4 highlights that for eight failure states, indicated as (1,1,1,1), the prescribed actions and corresponding value functions are identical. This consistency suggests a uniform strategy when the system is in this particular state. However, for other states, a clear trend emerges: the worse the system’s overall condition, the greater the likelihood of implementing component-level replacements. In Group 3 of Table 4, the actions indicate that the frequency of the component replacement action—denoted by 1—occurs more often compared to actions in Group 1.

The increasing property of the value functions is further illustrated by the results presented in Tables 3 and 4, which reveal clear trends in the data. For instance, in Group 1 of Table 3, the value functions for states that are deemed worse than the reference state (1,2,2,1)—specifically, states (1,1,1,1), (1,1,2,1) and (1,2,1,1)—exhibit lower values compared to that of state (1,2,2,1). Conversely, the value functions for states that are superior to state (1,2,2,1) are higher, confirming the expected increasing trend. In contrast, certain states resist straightforward classification, leading to irregularities in the value functions. For example, in Group 3 of Table 4, the value functions for states such as (3,1,2,1), (3,1,3,1), (3,1,3,2), (3,3,1,1), and (3,3,1,2) are all lower than that of state (3,2,1,3). On the other hand, the value functions for states like (3,1,3,3), (3,3,1,3), (3,3,2,2), and (3,3,3,1) are greater than that of state (3,2,1,3).

### 6.2. Analysis on the comparison results

As for the comparison models, their value functions for stage 1 (the expected costs over one year) under various system states are listed in Table 5. The results of Table 3 and 5 indicate that, for any system states, the alternate replacement strategy is the most economical and the expected cost over one year for comparison 2 is the highest. Furthermore, the costs for the alternate replacement strategy and comparison 1 are nearly the same and the cost difference between the alternate replacement strategy and comparison 2 are relatively higher. Based on the above analysis, under such parameter values in Tables 1 and 2, the advantage of the alternate replacement strategy mainly comes from the dynamic optimization of the replacement actions of component level.

**Table 5:** The value functions of comparative policies.

States	${V_{1}}^{\prime }$	${V_{1}}^{\prime \prime }$	States	${V_{1}}^{\prime }$	${V_{1}}^{\prime \prime }$
(1,1,1,1)	504.726	506.678	(2,2,2,3)	844.931	883.331
(1,1,1,2)	535.599	539.349	(2,2,3,1)	724.082	750.561
(1,1,1,3)	662.290	673.585	(2,2,3,2)	775.764	808.352
(1,1,2,1)	538.492	542.409	(2,2,3,3)	944.016	1081.124
(1,1,2,2)	576.239	583.081	(2,3,1,1)	701.606	721.222
(1,1,2,3)	722.024	742.730	(2,3,1,2)	755.581	781.789
(1,1,3,1)	621.463	629.368	(2,3,1,3)	943.186	1056.483
(1,1,3,2)	666.125	680.120	(2,3,2,1)	782.729	816.724
(1,1,3,3)	824.198	895.570	(2,3,2,2)	830.517	868.108
(1,2,1,1)	534.628	538.355	(2,3,2,3)	1002.912	1135.386
(1,2,1,2)	570.065	576.071	(2,3,3,1)	888.149	1017.201
(1,2,1,3)	710.310	727.365	(2,3,3,2)	929.792	1066.269
(1,2,2,1)	575.715	582.691	(2,3,3,3)	1374.726	1376.678
(1,2,2,2)	617.049	627.123	(3,1,1,1)	618.373	626.089
(1,2,2,3)	776.862	804.583	(3,1,1,2)	661.769	675.827
(1,2,3,1)	665.865	680.203	(3,1,1,3)	811.589	887.179
(1,2,3,2)	714.369	735.177	(3,1,2,1)	663.672	680.021
(1,2,3,3)	875.198	978.164	(3,1,2,2)	720.662	746.529
(1,3,1,1)	649.485	659.706	(3,1,2,3)	894.814	1029.624
(1,3,1,2)	696.586	712.246	(3,1,3,1)	740.314	807.538
(1,3,1,3)	874.026	940.467	(3,1,3,2)	796.153	903.267
(1,3,2,1)	707.631	727.194	(3,1,3,3)	1374.726	1376.678
(1,3,2,2)	761.014	787.138	(3,2,1,1)	661.219	675.628
(1,3,2,3)	946.907	1061.319	(3,2,1,2)	709.092	730.484
(1,3,3,1)	808.079	875.708	(3,2,1,3)	863.927	971.805
(1,3,3,2)	857.572	954.707	(3,2,2,1)	723.609	750.759
(1,3,3,3)	1374.726	1376.678	(3,2,2,2)	772.264	808.561
(2,1,1,1)	535.884	539.664	(3,2,2,3)	938.458	1081.430
(2,1,1,2)	572.882	579.625	(3,2,3,1)	800.295	913.586
(2,1,1,3)	716.160	736.941	(3,2,3,2)	850.375	984.697
(2,1,2,1)	575.402	582.843	(3,2,3,3)	1374.726	1376.678
(2,1,2,2)	622.265	633.928	(3,3,1,1)	795.157	866.790
(2,1,2,3)	795.461	829.746	(3,3,1,2)	845.926	947.783
(2,1,3,1)	663.779	679.878	(3,3,1,3)	1374.726	1376.678
(2,1,3,2)	721.115	746.341	(3,3,2,1)	881.764	1017.614
(2,1,3,3)	901.137	1029.233	(3,3,2,2)	924.077	1066.599
(2,2,1,1)	572.202	579.078	(3,3,2,3)	1374.726	1376.678
(2,2,1,2)	613.298	623.292	(3,3,3,1)	1374.726	1376.678
(2,2,1,3)	771.574	799.393	(3,3,3,2)	1374.726	1376.678
(2,2,2,1)	623.587	635.735	(3,3,3,3)	1374.726	1376.678
(2,2,2,2)	668.344	683.875			

### 6.3. The effect of system parameters on the value functions

To gain more insights into the model, the effect of some system parameters on the value function $V_{1}$ is checked in this section. Unless otherwise specified, in the following process of analysis, when one parameter changes, the values of the other parameters remain the same as in 5.1.

**Fig 5:**
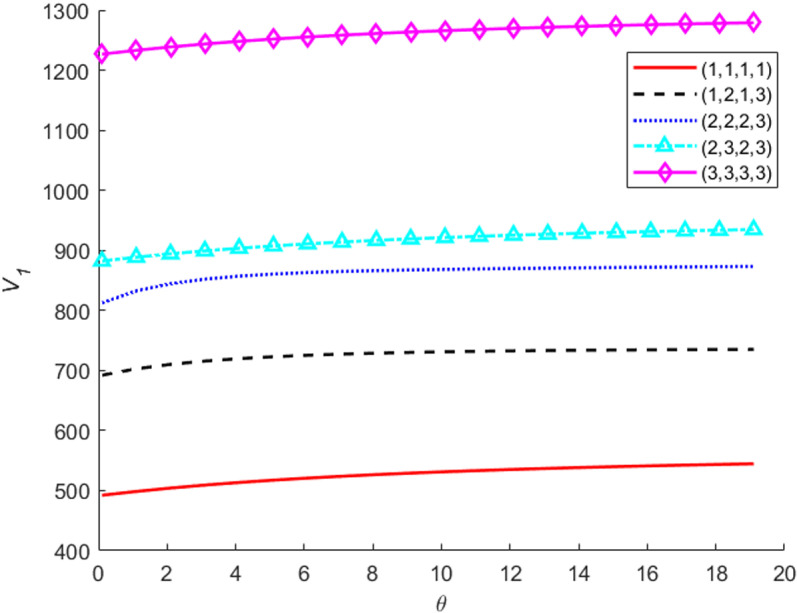
The value functions for different dependence strength.

Let the parameter for the dependence strength θ change from 0 to 20 by step 1, the value functions $V_{1}$ for the different system states are sketched in Fig 5. The results indicate that: (a) given a system state, the value functions increase as the dependence strength increases. The main reason for the above trend is as follows: when the dependence strength gets greater, the probability that the common-mode deterioration among components will get larger, subsequently, system degradation process will get faster and the probabilities of system or component failures over the four stages will increase. (b) The value functions for the states at an intermediate level are more sensitive to the dependence strength. The trend may be related to the copula function we have chosen. Degradation increment processes with Clayton-Copula are lower tail dependence, that is they are interdependent strongly with each other when their degradation states are relatively good. While the value functions are costs incurred when the system is in worse states, therefore the value functions for better states (state (1,1,1,1), state (1,2,1,3) are almost insensitive to the dependence strength. As for these poor states (state (3,3,3,3), state (2,3,2,3), the interdependency of them is relatively weak. Furthermore, the system can be replaced in entirety and then transit to the best state and every component can be also replaced separately. On account of the above two reasons, the value functions are also almost insensitive to the dependence strength. For the system states at an intermediate level, the interdependency of components is relatively strong and the replacement actions can be chosen with a higher probability, thus, the value functions are more sensitive to the dependence strength.

**Fig 6:**
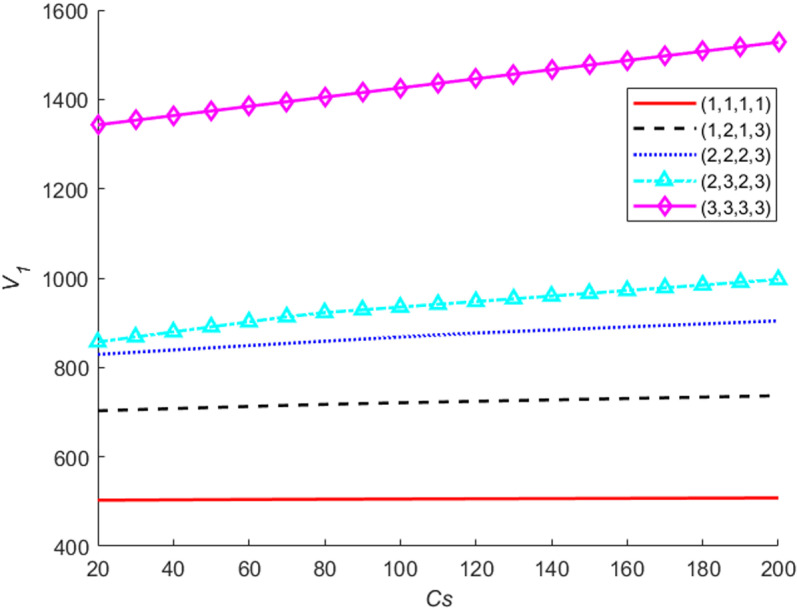
The value functions for different set-up costs.

Let the set-up cost $C_{s}$ change from 20 to 200 by step 10, the value functions $V_{1}$ for the different system states are presented in Fig 6. The results indicate that: (a) when the system is in the best state (1,1,1,1), the set-up cost has almost no impact on the value function. For the state (1,1,1,1), the optimal actions under the four stages are doing nothing and there is no set-up cost, thus the value function is invariant for different set-up costs. (b) For the other states, the worse the system states and the larger the set-up cost, the lager the value functions are. An intuitive explanation is as follows: when the system states get worse, the likelihood of the replacement actions will increase, hence, the value functions are larger and also more sensitive to the changing of the set-up costs.

**Fig 7:**
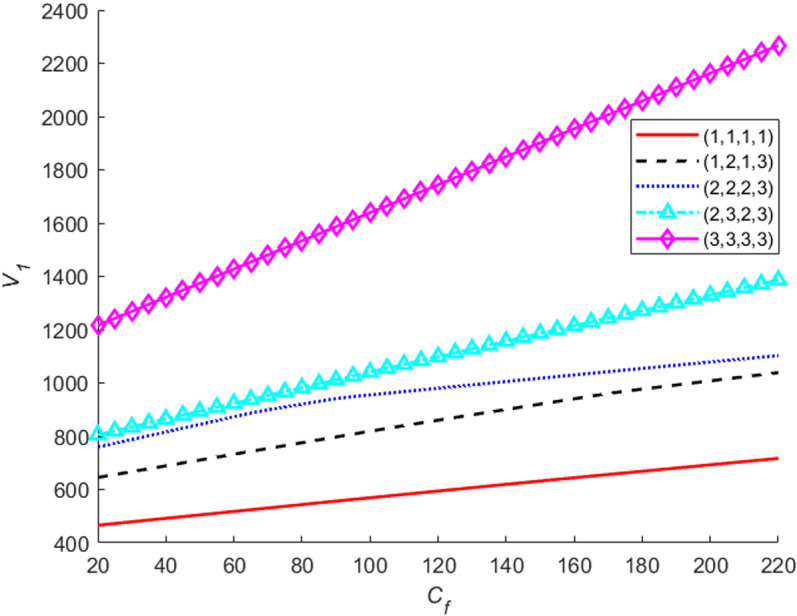
The value functions for different fundamental failure penalty costs.

Let the fundamental failure penalty cost of system failures change from 20 to 200, the value functions $V_{1}$ for the different penalty cost are sketched in Fig 7. The figure indicates that: (a) when the system is in the best state, the cost has a little impact on the value function. The main reason is that for the best state, the system failure probabilities are very small under the four stages. (b) For the other states, the worse the system states and the larger the penalty cost, the lager the value functions are. The main reason for the above trend is as follows: the worse the system states, the higher probabilities of system failures are and hence the value functions increase as the fundamental penalty costs increase.

## 7. Conclusions

Based on the operational and maintenance practices for steel rails, this study constructs a *k*-out-of-*n*: F system model that incorporates common-mode degradation, alongside a dynamic and alternate replacement strategy addressing both system-level and component-level interventions. The dependence structure of the system is effectively characterized using a copula function, providing a robust framework for analyzing relationships between components. This strategy serves as a valuable guide for the annual maintenance and repair planning of railway lines. To derive the optimal maintenance strategy, a MDP model consisting of four stages is established. The model includes analytical expressions for the discretized state transition matrix pertinent to the multiple-component system, and an algorithm is designed to implement these expressions efficiently. The monotonicity of the value functions is verified through the application of mathematical induction, ensuring the reliability of the model’s outcomes. A numerical example is presented to demonstrate the effectiveness of the proposed model and validate the accuracy of the conclusions drawn. The results of this numerical example indicate that, under the Clayton Copula structure, value functions increase as the dependence strength, set-up cost, and fundamental failure penalty cost rise. Furthermore, it is noteworthy that states positioned at an intermediate level exhibit greater sensitivity to changes in dependence strength.

The common-mode deterioration phenomenon can be found in many fields, such as electronic engineering, mechanical engineering, structural engineering, automotive engineering, aerospace engineering and so on. The modeling method and maintenance strategy proposed in this paper are helpful in reliability assessment and optimizations of maintenance strategies.

For future research, the modeling and optimization of replacement activities for a group of steel rail grids is recommended. For a group of steel rail grids, diverse maintenance decisions, such as group maintenance, opportunity maintenance, are possible and the maintenance decisions of a group of steel rail grids are also practical. Moreover, the modeling and maintenance optimization of steel rail grids with hierarchical stochastic dependency deserve further investigation.
